# Physicians on the Canadian Border

**Published:** 1895-07

**Authors:** 


### Editorial, Etc.

PHYSICIANS ON THE CANADIAN BORDER.—Counsel Twitchell, of Kingston, Canada, writes to the Department March 27, 1895:

"I am informed through the public press—the information being verified by letter—that physicians living south of the Canadian boundary have recently been prohibited from attending patients living in Canada. I presume that the ostensible reason for this prohibition must be the alleged inferiority ot the medical education of these practitioners and the consequent danger to the Canadian people. Of the professional competency of the physicians on the American side of the border, I know but little, but the indifference of some of the State governments in the United States to the qualifications of their medical practitioners is in marked contrast with the action of the Province of Ontario. For a number of years the United States seems to have been the dumping ground for medical students who did not apply for or failed to pass the examination required to qualify them to practice in Ontario. If this matter were fully understood by our State governments, I have thought that the instinct of self-protection, combined with State and national pride would cause them to require as high a standard of proficiency to practice medicine in their States as is required in Canada. During the year 1894, one hundred students graduated from the different medical colleges of Ontario. Of these, fifty-three passed the examination required by the medical council for the practice of medicine in Canada, leaving forty seven unaccounted for. I think I can safely venture the opinion that if the passage of the examination of the medical council had been required to practice in the United States, forty-seven more medical students would have fitted themselves for the examination."—U. S. Consular Reports, May, 1895.

